# Combined spatiotemporal and frequency-dependent shear wave elastography enables detection of vulnerable carotid plaques as validated by MRI

**DOI:** 10.1038/s41598-019-57317-7

**Published:** 2020-01-15

**Authors:** David Marlevi, Sharon L. Mulvagh, Runqing Huang, J. Kevin DeMarco, Hideki Ota, John Huston, Reidar Winter, Thanila A. Macedo, Sahar S. Abdelmoneim, Matilda Larsson, Patricia A. Pellikka, Matthew W. Urban

**Affiliations:** 1 Department of Biomedical Engineering and Health Systems, KTH Royal Institute of Technology, Stockholm, Sweden; 2 Department of Clinical Sciences, Karolinska Institutet, Stockholm, Sweden; 3 Department of Cardiovascular Medicine, Mayo Clinic College of Medicine, Rochester, MN United States of America; 4 Division of Cardiology, Dalhousie University, Halifax, Nova Scotia Canada; 5 Department of Radiology, Walter Reed National Military Medical Center, Bethesda, MD United States of America; 6 Department of Radiology, Uniformed Services University of Health Sciences, Bethesda, MD United States of America; 7 Department of Diagnostic Radiology, Tohoku University Hospital, Sendai, Japan; 8 Department of Radiology, Mayo Clinic College of Medicine, Rochester, MN United States of America; 9 Department of Molecular Medicine and Surgery, Karolinska Institutet, Stockholm, Sweden

**Keywords:** Translational research, Biomedical engineering

## Abstract

Fatal cerebrovascular events are often caused by rupture of atherosclerotic plaques. However, rupture-prone plaques are often distinguished by their internal composition rather than degree of luminal narrowing, and conventional imaging techniques might thus fail to detect such culprit lesions. In this feasibility study, we investigate the potential of ultrasound shear wave elastography (SWE) to detect vulnerable carotid plaques, evaluating group velocity and frequency-dependent phase velocities as novel biomarkers for plaque vulnerability. In total, 27 carotid plaques from 20 patients were scanned by ultrasound SWE and magnetic resonance imaging (MRI). SWE output was quantified as group velocity and frequency-dependent phase velocities, respectively, with results correlated to intraplaque constituents identified by MRI. Overall, vulnerable lesions graded as American Heart Association (AHA) type VI showed significantly higher group and phase velocity compared to any other AHA type. A selection of correlations with intraplaque components could also be identified with group and phase velocity (lipid-rich necrotic core content, fibrous cap structure, intraplaque hemorrhage), complementing the clinical lesion classification. In conclusion, we demonstrate the ability to detect vulnerable carotid plaques using combined SWE, with group velocity and frequency-dependent phase velocity providing potentially complementary information on plaque characteristics. With such, the method represents a promising non-invasive approach for refined atherosclerotic risk prediction.

## Introduction

Atherosclerotic disease is recognized as a diffuse systemic vascular process, and rupture and erosion of carotid atherosclerotic plaques is the number one cause of cerebrovascular mortality in the world^1^. Fatal plaque-related events however often arise without prior symptoms^2^, and the task of plaque risk stratification – identifying rupture-prone vulnerable plaques from stable rupture-resistant phenotypes – is of pivotal clinical importance. Surgical endarterectomy is recommended on the basis of carotid stenosis^3^, however it has been extensively shown that vulnerable plaques are rather identified by their composition than on their extent of luminal obstruction^4,5^. Several studies have shown that in the majority of patients with acute ischemic syndromes culprit coronary sites had less than 50–70% diameter narrowing, and plaques producing non-flow-limiting stenosis accounted for more cases of plaque rupture than plaques with more severe stenosis^6,7^. Instead, the rupture risk was found to be determined by plaque composition rather than plaque size or degree of stenosis^8^. In particular, plaques with a thin fibrous cap covering a lipid-rich necrotic core (LRNC) (thin fibrous cap atheroma, TFCA) and plaques with pronounced neovascularization and an active center with intraplaque hemorrhage (IPH) or juxtaluminal thrombus have been identified as particularly rupture prone^4,5^.

For the *in-vivo* characterization of atherosclerotic plaques, a number of imaging-based techniques have been proposed. Catheter-based methods have shown to be able to detect vascular lipid accumulations^9,10^, however are limited by their inherent invasiveness. High calcium-sensitivity with computed tomography (CT), or excellent soft-tissue contrast by magnetic resonance imaging (MRI) has also been utilized for plaque characterization^11,12^. However, these modalities are limited by use of ionizing radiation (CT) or high monetary expense and limited availability (MRI). Non-invasive ultrasound imaging represents a real-time, inexpensive, and widely available option, with the technique already used in the general screening of cardiovascular disease and evaluation of carotid stenosis^13^.

One emerging field of particular interest is non-invasive ultrasound elastography, where mapping of tissue mechanical properties can be achieved by tracking the constitutive response following regional tissue displacement. With a range of constitutive biomarkers proposed (strain, shear wave velocity, shear modulus), ultrasound elastography holds particular promise for refined plaque characterization, with mechanical properties shown to vary significantly between fibrous, fibroatheromatous, and atheromatous plaques^10,14,15^, or even between symptomatic and asymptomatic plaques^16,17^. However, the spatial confinements and complex composition of atherosclerotic plaque affects accuracy^18,19^, and *in-vitro* studies have shown that combined spatiotemporal and frequency-dependent shear wave analysis might be required for accurate plaque differentiability^18^. However, such analysis has yet to be evaluated *in-vivo*.

The aim of this study was therefore to, for the first time, apply combined spatiotemporal and frequency-dependent ultrasound shear wave elastography (SWE) to non-invasively assess atherosclerotic plaque characteristics *in-vivo*. Specifically, group velocity and phase velocity SWE were assessed on a carotid plaque cohort, evaluating and validating output against reference MRI.

## Methods

All subjects participated under informed consent, with the study approved by the Mayo Clinic Institutional Review Board (Protocol 14-000555) and with all methods performed in accordance with relevant guidelines and regulations. The inclusion criteria were adults age ≥18 years, suspected or known coronary artery disease and referral for stress echocardiography at Mayo Clinic. The exclusion criteria were previous carotid surgery or angioplasty, prior contraindication to ultrasound contrast agents (as such were used in a separate study), pregnancy, or renal dysfunction.

### Study population

**Figure 1:**
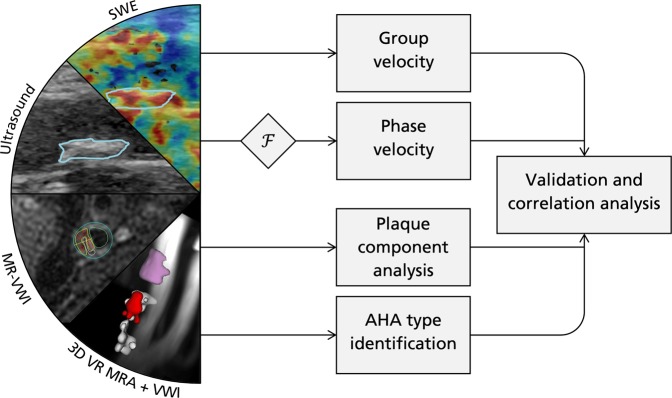
Overview of methodological setup. Ultrasound B-mode and SWE imaging is performed, providing both spatiotemporal group velocity (time-domain) and frequency-dependent phase velocity (after Fourier transform, ${\boldsymbol{ {\mathcal F} }}$). Velocities are validated and correlated with MRI-derived plaque components, as well as AHA type classification. For the ultrasound and SWE panels, the plaque is delineated in blue. In the MR-vessel wall imaging (VWI) panel, one of the 2D black-blood VWI series is displayed with the analysis from MRI PlaqueView superimposed including the lipid-rich necrotic core (LRNC, yellow), intraplaque hemorrhage (IPH, red), calcifications (CA, white), loose matrix (purple), and vessel outline (blue). In the 3D volume rendering (VR) MR angiography (MRA) + VWI panel, the detected plaque components are superimposed on the MRA and displayed as a volume rendered image using the same color scheme.

Twenty-two subjects presenting for stress echocardiography and found to have carotid atherosclerotic plaque detected by conventional ultrasound imaging were enrolled. In total, 29 plaques were identified (17 in the right internal, 12 in the left internal carotid artery). SWE and MRI of each plaque were performed within 4 weeks. Due to technically unsuccessful MRI, two plaques were excluded from the study. Characteristics of the remaining subjects are provided in Table 1. An overview of the methodological setup is given in Fig. 1.

**Table 1:** Subject characteristics and risk factors.

Demographics and risk factor	Mean ± SD or number of subjects, n	Range (if applicable)
Age, yrs	67.8 ± 8.4	45–84
Male, n	16 (80%)	
BMI, kg/m^2^	30.6 ± 4.5	22.1–38.3
Hypertension, n	12 (60%)	
History of cardiovascular disease, n	12 (60%)	
Coronary artery disease, n	8 (40%)	
Stroke, n	4 (20%)	
Transient ischemic attack, n	1 (5%)	
**Smoking status**		
Active, n	4 (20%)	
Prior, n	5 (25%)	
Never smoked, n	11 (55%)	
Hyperlipidaemia, n	17 (85%)	
Total cholesterol, mg/dL	170.1 ± 41.3	120–263
Triglycerides (TG), mg/dL	175.3 ± 107.7	50–420
HDL cholesterol, mg/dL	46.9 ± 14.3	23–80
LDL cholesterol, mg/dL	89.1 ± 37.3	48–170
**Medication**		
Aspirin, n	15 (75%)	
Statins, n	18 (90%)	
Nitrates, n	8 (40%)	
Beta blockers, n	12 (60%)	
Calcium channel blockers, n	5 (25%)	
ACEI, n	8 (40%)	
**Carotid stenosis**		
Severe (>70%), n	1 (5%)	
Moderate (50–69%), n	3 (15%)	
None/Mild (<50%), n	16 (80%)

### Ultrasound shear wave elastography

#### Imaging protocol

Ultrasound SWE was performed on all plaques using a General Electric Logiq E9 system (GE Healthcare, Wauwatosa, WI, USA) with a 9 L linear array transducer and with ∼5–15 acquisitions retrieved in the longitudinal (L) and transverse (T) imaging views, respectively, for each plaque. For each acquisition, the plaque was centered inside a manually positioned imaging window, with the cross-section having the most significant stenosis chosen as the representative view for each plaque. If such a view was obstructed by excessive shadowing from intraplaque calcifications, a neighboring cross-section was chosen. For the SWE, a dual-sided acoustic radiation force push was used, utilizing two focused push beams simultaneously triggered at the left- and right-hand sides of the region-of-interest (ROI), respectively (similar to the setup in Song *et al*.^20^). A push frequency of 4.1 or 5.0 MHz was utilized with a push duration of 400 μs, followed by time-aligned sequential acquisition (frequency: 5 MHz)^21^. From each acquisition, in-phase/quadrature (IQ) data was exported, at a resolution of approximately 0.30 mm^2^ and with recordings lasting for around 18 ms each.

#### Shear wave elastography analysis

The plaque border ROI was visually identified in the B-mode images and used as a segmentation mask for the data processing. Axial particle velocities were derived in the entire field of view using a 2D autocorrelation algorithm^22^, using directional filtering to identify leftward and rightward travelling shear wave, respectively. For the plaque ROI, depth-averaged velocities were estimated in the masked data. A set of refined velocity parameters was retrieved, specifically in the form of group and phase velocity (for technical details of the same protocol, see^18^).

Group velocity. The group velocity, the spatiotemporal wave velocity of the wave packet traveling in time-domain, was acquired from the axial particle velocity maps. Group velocity represents the conventional output metric utilized in clinical and research-based ultrasound elastography.

Group velocity was estimated from calculated axial particle velocity maps using a linear fit time-to-peak (TTP) method^23^ with a random sample consensus filter^24^ added to improve result quality. The quality-of-fit was evaluated by the ratio of output inliers. If this did not exceed 50%, or if the linear fit was visually assessed as not pertaining to the main wave, manual cropping of the axial particle velocity map was introduced, within which a new TTP estimate was retrieved.

Phase velocity. With shear wave propagation in confined elastic plaque media giving rise to frequency-dependent dispersion behavior^19,25^, assessment of phase velocity was also performed. A two-dimensional fast Fourier transform was employed on the axial particle velocity maps, with discrete phase velocities identified as the velocity at which the intensity was maximized in Fourier space at a given frequency. To generate discrete metrics, phase velocities were averaged within ranges of 200–300 Hz, 300–400 Hz, and 400–500 Hz, respectively.

The quality of the dispersion data was visually assessed, with data cropped to only contain information from the fundamental zero-order mode. Dispersion data deemed to be overly corrupted by noise or distinctly skewed by higher-order wave modes – both in the lower and upper frequency ranges –were discarded.

### Magnetic resonance imaging

#### Imaging protocol

Carotid bifurcation MRI was performed at 3 T with a carotid phase-array surface 6-channel coil (Neocoil LLC, Pewaukee, WI, USA) using a previously described multi-contrast protocol^26^. In brief, the sequences included 3D time-of-flight MR angiography (TOF-MRA), a 3D black-blood sequence optimized to detect IPH (magnetization-prepared rapid acquisition gradient echo, MPRAGE), 2D pre-contrast black-blood T1 weighting (T1W), 2D T2 weighting (T2W), and contrast-enhanced 2D black-blood T1W (CE-T1W). Each sequence was acquired as axially stacked images centered on the carotid bifurcation (in-plane resolution: 0.625 mm^2^, slice thickness: 1 or 2 mm for 3D or 2D sequences, respectively).

#### MRI analysis and plaque classification

Two experienced reviewers (J.K.D. and H.O.) who were blinded to the SWE and clinical findings reviewed all MR images and reached a consensus decision for each plaque feature using a previously described protocol^27^.

For processing, the carotid bifurcation was used as landmark to match the MR series. Manual segmentation of the lumen and outer wall was performed using a dedicated image analysis tool (MRI-PlaqueView; VP Diagnostics, Seattle, WA, USA)^28,29^. Plaque components such as LRNC, fibrous cap, IPH, loose matrix, and calcification were identified and volume measurements provided using previously published and histologically validated criteria^30,31^. In addition to total component volume, percent volume (component volume/total plaque volume) and percent area (component area/wall area, given in the axial image with largest percent area of the component) were calculated. The thickness of the fibrous cap was defined as the region between lumen and delineated LRNC. Mean, maximum, and minimum cap thickness, cap length, and cap volume were also reported. All identifications were based on general criteria where: a) LRNC without hemorrhage appears as hypointense on T2W and CE-T1W, and isointense on T1W- and TOF-images, b) IPH appears as hyperintense on TOF-, T1W-, and MPRAGE-images, c) calcifications appear as hypointense on all images, d) loose matrix appears as hyperintense on T2W and CE-T1W, and e) plaque fibrous cap was identified as separating lumen and LRNC on TOF and CE-T1W.

In addition to this, each plaque was assigned a type classification following the modified American Heart Association (AHA) classification of carotid atherosclerotic plaques^12^, where type scores are associated with plaque phenotype. In brief, AHA type I-II plaque shows near-normal wall thickness and no calcifications. AHA type III plaque demonstrates diffuse intimal thickening or small eccentric plaque with no calcifications. AHA type IV-V demonstrates a lipid or necrotic core surrounded by fibrous tissue with possible calcifications. AHA type VI is a complex plaque with possible surface defect, IPH or juxtaluminal thrombus. AHA type VII plaque is a calcified plaque. AHA type VIII is a fibrotic plaque without lipid core and with possible small calcifications. Importantly, the AHA type VI plaques are associated with a rupture-prone morphology, with the typical hallmarks of a vulnerable plaque (LRNC, TFCA, IPH) all potentially present within this complex lesion score.

### Data evaluation and statistical analysis

From each plaque, SWE velocities were quantified as mean ± standard deviation before being used in any further cohort-pooled analysis.

Group and phase velocity data were evaluated as a function of AHA type, with differences inferred using a two-sided Wilcoxon rank sum test (significance at p < 0.05). The analysis was separated for imaging plane (L- and T-view) and velocity metric (group velocity, and discrete phase velocity), respectively.

Correlations between SWE velocities and MRI-quantified plaque metrics were evaluated by calculating the Pearson and Spearman correlation coefficients for each plaque metric mean, respectively (correlation set at |R| ≥ 0.5 and p < 0.05). In the case that a variable could not be defined for an individual plaque, the plaque was excluded from that particular correlation analysis.

Correlations between SWE velocities and blood lipid levels (high-density lipoprotein (HDL), low-density lipoprotein (LDL), triglycerides (TG), and total cholesterol) were also assessed in a similar fashion.

All evaluations were performed within MATLAB R2016a (MathWorks, Natick, MA, USA).

### Disclaimer

The views expressed in this article are those of the authors and do not reflect the official policy of the Department of Army/Navy/Air Force, Department of Defense, or U.S. Government.

## Results

From the 27 plaques, 410 SWE acquisitions were performed. Out of these, 28 were excluded from the group velocity analysis due to poor signal quality. For the phase velocity analysis, an additional 64/70/116 acquisitions were excluded at 200–300/300–400/400–500 Hz, respectively, due to loss of signal quality or interfering higher order modes. For a single plaque assessment, the real-time SWE acquisition was followed by an offline analysis stage where plaque ROI, and data quality control were performed. However, once passed, all SWE metrics could be derived within <1 min.

**Figure 2:**
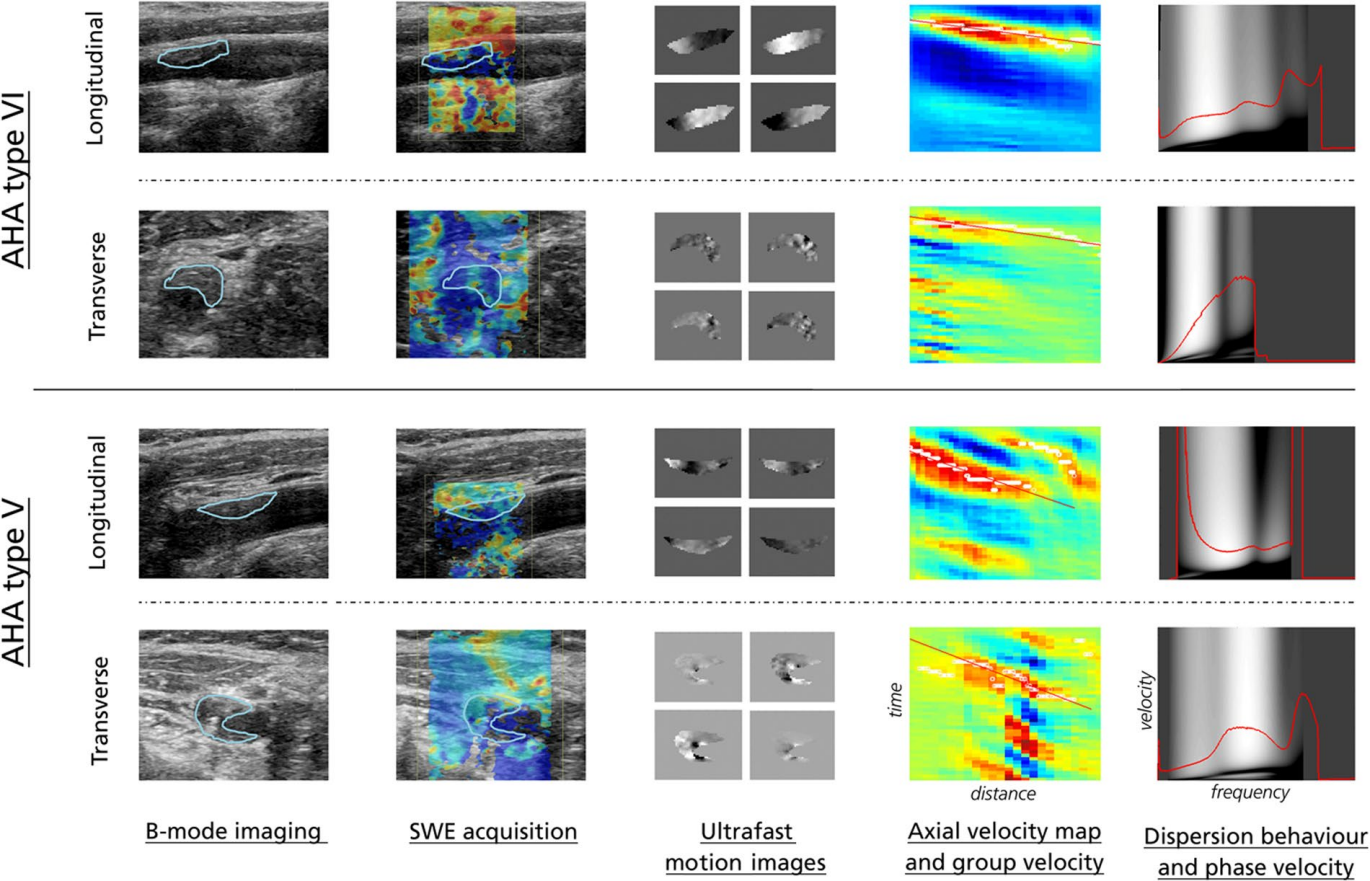
Overview of generated SWE images. From left to right: Ultrasound B-mode images, SWE acquisition, ultrafast motion images obtained from data autocorrelation (four snapshot motion images displayed, from upper left to lower right), axial velocity map (space-time domain) with TTP-estimated group velocity (red slope), and Fourier-generated dispersion behavior and phase velocity map (velocity-frequency domain). Examples are given in longitudinal and transverse view for one AHA type VI, and one AHA type V plaque, respectively. Note that for the dispersion curves, data was discarded in frequency-ranges where corruption from noise or higher-order modes seemed visually apparent (in this case, lower frequency-range data for the AHA type V plaque in the longitudinal view plan was discarded from further analysis, as was higher frequency-range data for the AHA type VI plaque in transverse view). For all other presented cases, the 200–500 Hz range was deemed appropriate for inclusion. All examples are shown over a frequency range of 0–750 Hz.

An example of the generated SWE data is given in Fig. 2.

### SWE velocities and plaque AHA type

**Figure 3:**
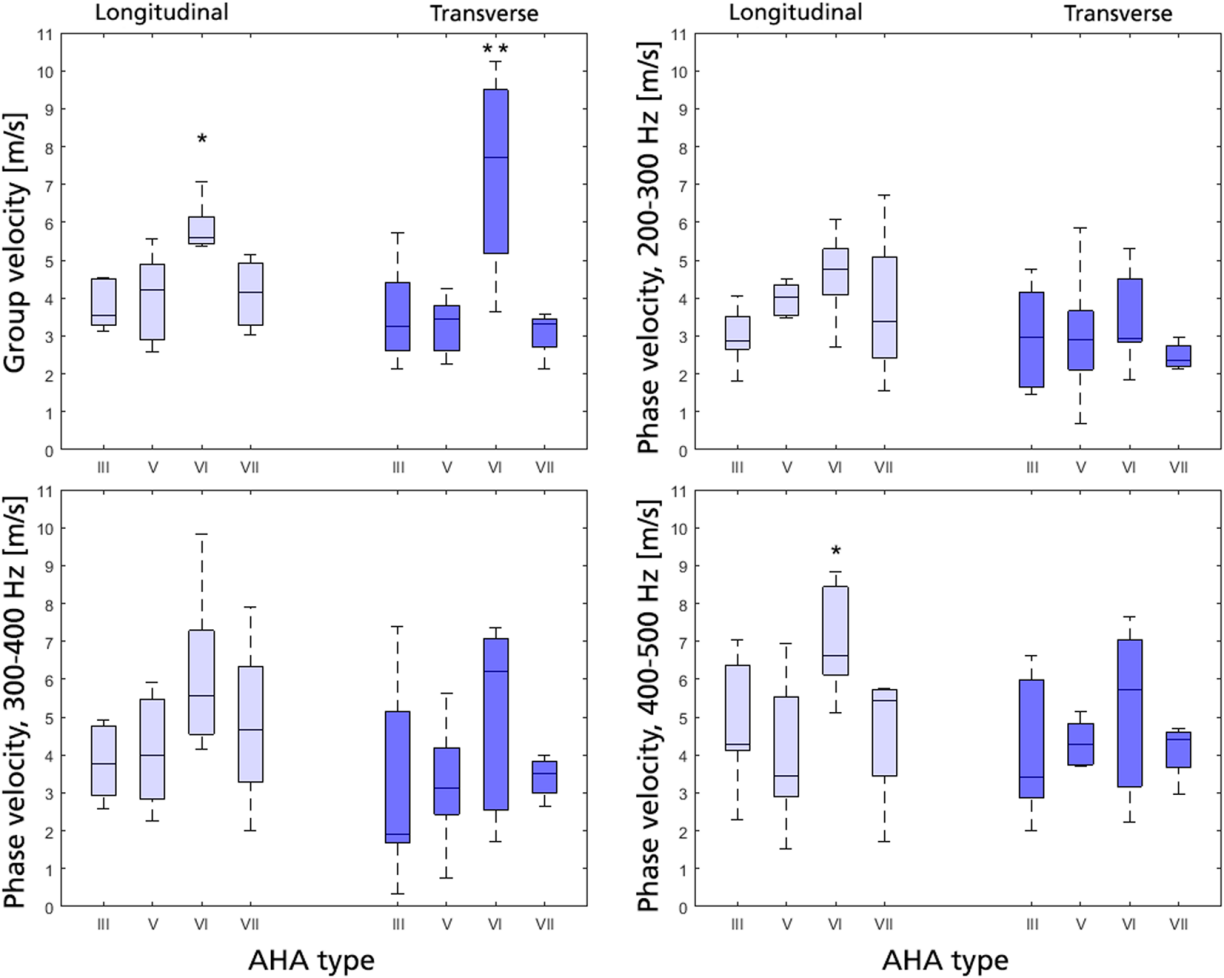
SWE velocities, classified according to AHA type. Data sorted in longitudinal (light blue) and transverse view (dark blue), shown as group velocity (top left), and phase velocity at 200–300 Hz (top right), 300–400 Hz (bottom left) and 400–500 Hz (bottom right), respectively. Significance against all other groups is given as *(p < 0.05) or **(p < 0.01).

Of the 27 imaged plaques, 7 were classified as AHA type III, 8 as type V, 8 as type VI and 4 as type VII. Group and phase velocities are shown graphically in Fig. 3, with mean ± standard deviation provided for each AHA type in Table 2. Note that the SWE standard deviation for an individually assessed plaque was around 1–2 m/s over the entire cohort and for group and phase velocity, respectively.

**Table 2:** SWE velocities (m/s) grouped according to AHA type. Data given as mean ± standard deviation.

Velocity type	Imaging plane	Type III	Type V	Type VI	Type VII
Group vel	L	4.2 ± 1.1	4.0 ± 1.1	5.8 ± 0.6*	4.1 ± 1.0
	T	3.6 ± 1.3	3.3 ± 0.7	7.3 ± 2.5**	3.1 ± 0.6
Phase vel 200–300 Hz	L	3.0 ± 0.7	4.0 ± 1.1	4.6 ± 1.1	3.8 ± 2.2
	T	3.0 ± 1.3	3.0 ± 1.5	3.5 ± 1.2	2.5 ± 0.4
Phase vel 300–400 Hz	L	3.8 ± 1.0	4.1 ± 1.5	6.1 ± 2.1	4.8 ± 2.4
	T	3.3 ± 2.5	3.2 ± 1.5	5.0 ± 2.5	3.4 ± 0.6
Phase vel 400–500 Hz	L	4.8 ± 1.7	4.1 ± 1.9	7.0 ± 1.4*	4.6 ± 1.9
	T	4.1 ± 1.8	4.3 ± 1.4	5.2 ± 2.1	4.1 ± 0.8

*(p < 0.05) or **(p < 0.01) indicates statistical differentiation from all other plaque types at that velocity and imaging plane. L = longitudinal view, T = transverse view.

AHA type VI plaques showed significantly higher group velocity compared to all other plaque types, respectively, with a mean group velocity of 5.8 m/s (L) and 7.3 m/s (T) compared to 4.0–4.2 m/s (L) and 3.1–3.6 m/s (T), respectively (p < 0.02 and p < 0.004 for L/T). This effect diminished when applying frequency-dependent phase velocity analysis, however reappeared for the highest frequency band in L-view, where AHA type VI plaques showed significantly higher wave velocities (p < 0.04).

For the AHA classification, no other evaluated metric (age, sex, BMI, systolic or diastolic blood pressure, smoking status, blood lipid levels, medication, stenosis degree) could statistically differentiate the AHA type VI lesions from the rest of the evaluated plaque cohort.

### SWE velocities and intraplaque component correlations

**Figure 4:**
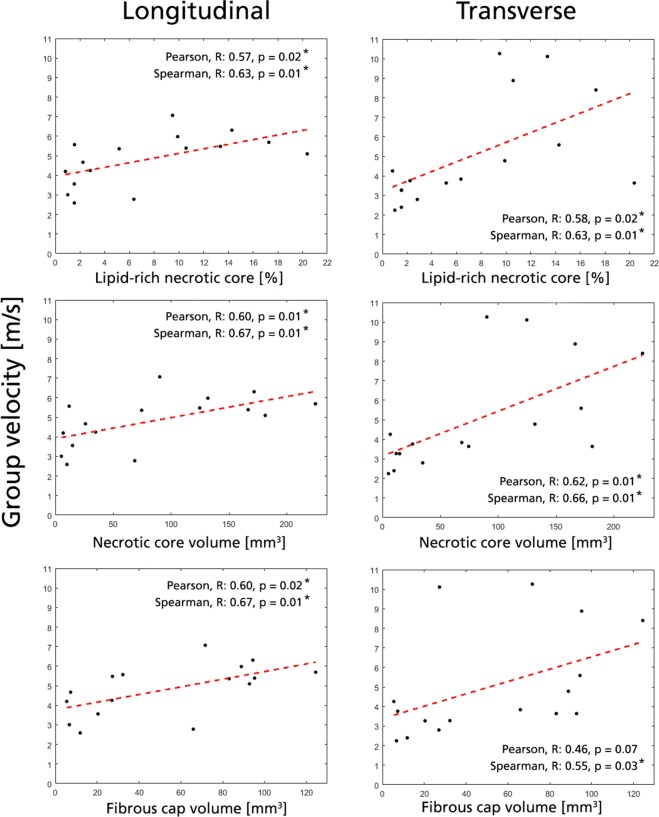
Selected significant correlations with group velocity. Data shown for longitudinal (left column) and transverse view (right column). All acquisitions for a given plaque is represented by their mean value (black dots), with an added Pearson linear regression line (red dashed). Significant correlations (*) are given for |R| > 0.50 and p < 0.05.

**Figure 5:**
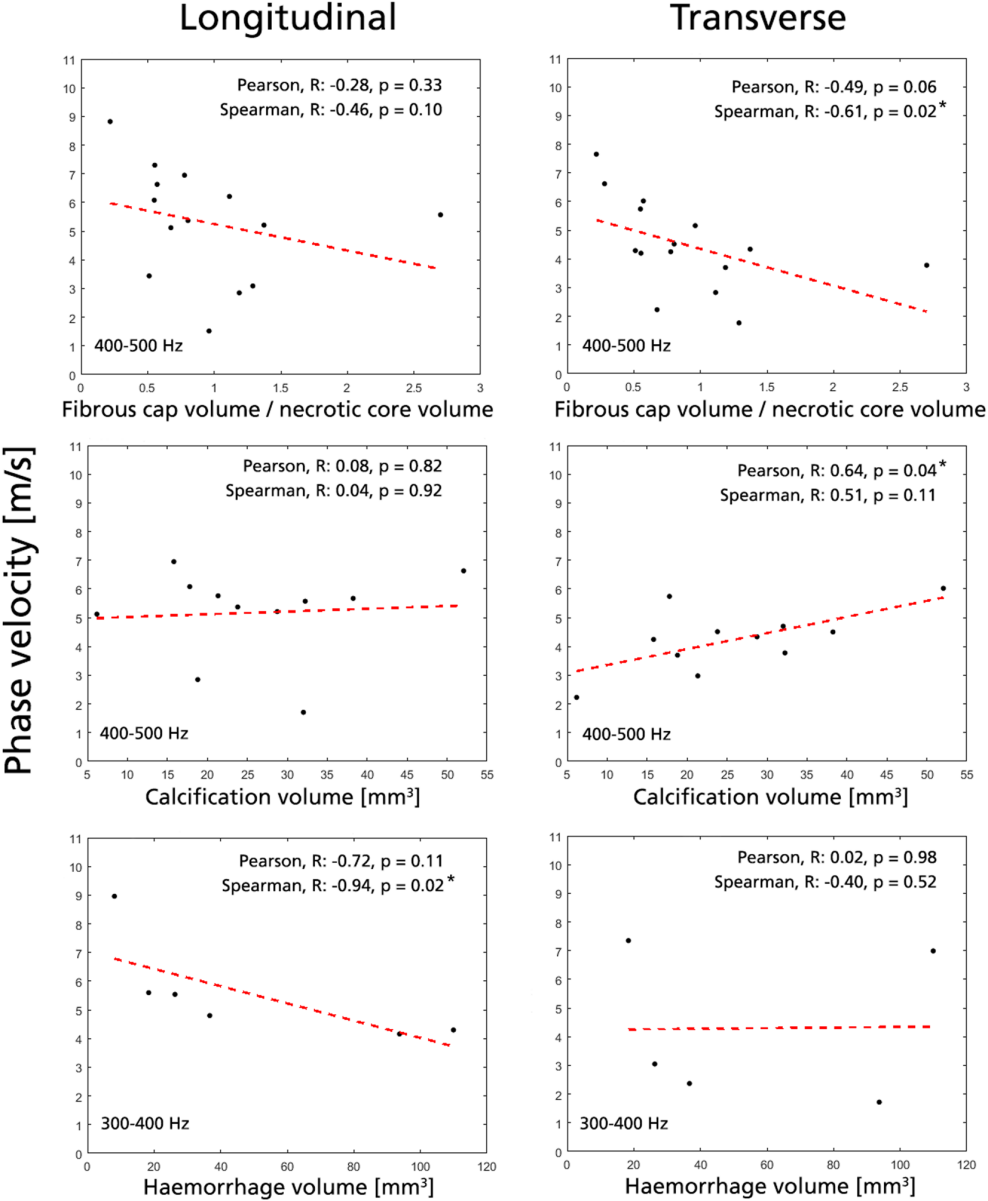
Selected significant correlations with phase velocity. Data shown for longitudinal (left column) and transverse view (right column). All acquisitions for a given plaque is represented by their mean value (black dots), with an added Pearson linear regression line (red dashed). Significant correlations (*) are given for |R| > 0.5 and p < 0.05.

Correlations for a few plaque parameters are provided in Figs. 4, 5, selected based on their correlation and importance for plaque stability. Complete correlation tables are provided in Supplementary Tables A–D.

For group velocity, positive correlation was seen with the amount of LRNC in both imaging planes (Pearson R = 0.57/0.58, p = 0.02/0.02 for L/T-view). Similarly, positive correlation (Pearson R = 0.60/0.62, p = 0.01/0.01 for L/T-view) was observed with necrotic core volume. Positive correlation in group velocity was observed with plaque cap volume, however only significant linear correlation was seen in L-view (Pearson R = 0.60/0.46, p = 0.02/0.07 for L/T-view). Conversely, no correlation could be seen with any plaque geometry metrics (length, thickness, or plaque area) or plaque cap dimensions.

For frequency-dependent phase velocity, the correlation strength increased with increasing frequency band with four unique correlations (|R| > 0.50, p < 0.05) given at 400–500 Hz and 300–400 Hz, respectively, compared to one at 200–300 Hz. At the highest frequency band, phase velocities in the T-view showed negative nonlinear rank correlation to the ratio between fibrous cap and necrotic core volume (Spearman R = −0.61, p = 0.02). T-view phase velocity at the highest frequency range also showed positive linear correlation with plaque calcification volume (Pearson R = 0.64, p = 0.04) and negative linear correlation with plaque loose matrix content (Pearson R = −0.64, p = 0.03). Additionally, plaque hemorrhage volume also indicated negative nonlinear rank correlation with longitudinal phase velocity in 300–400 Hz (Spearman R = −0.94, p = 0.02).

### SWE velocities and blood lipid correlations

For group velocity only T-view imaging correlated with blood lipid levels, with positive linear correlation observed with the amount of HDL (Pearson R = 0.56, p < 0.01).

For phase velocity, a similar positive linear correlation with HDL was observed in T-view, with results consistent over the entire frequency range (Pearson R = 0.59/0.63/0.54 with p =  < 0.01/ < 0.01/ < 0.01 for 200–300/300–400/400–500 Hz, respectively). Additionally a range of other correlations were observed at varying frequencies. Noteworthy, TG levels correlated linearly with T-view phase velocity at the highest frequency range (Pearson R = −0.52, p < 0.01), whereas the total non-HDL cholesterol level indicated negative linear correlation with T-view phase velocities at the lowermost frequency range (Pearson R = −0.64, p < 0.01).

## Discussion

This study implemented for the first time combined spatiotemporal and frequency-dependent ultrasound SWE analysis to non-invasively assess carotid atherosclerotic plaque characteristics *in-vivo*. The results show that identification of vulnerable lesions can be achieved using SWE, where AHA type VI plaques showed significantly higher group velocity, with similar trends indicated for high-frequency phase velocity. Additionally, a set of novel SWE-based biomarkers have been identified, with group velocity and phase velocity correlating with plaque LRNC content, fibrous cap/necrotic core volume ratio, and IPH volume; all entities associated with status of plaque stability.

### Significantly higher shear wave velocity in AHA type VI plaques

AHA type VI plaques showed significantly higher group velocity compared to any other evaluated AHA type, and similar differentiation was observed for phase velocity at the highest frequency band. With AHA type VI plaques identified as particularly rupture prone this is a central finding, underlining the potential of SWE for clinical plaque risk assessment. AHA type VI plaques do not necessarily correlate with stenosis degree^32^, and hence the proposed evaluation expands on current clinical guidelines for plaque risk stratification. With AHA type VI plaques representing a complex set of culprit lesions the explanation for the increased wave velocity is however difficult to isolate, and in fact contradictory results have been reported in literature. Collagenous and calcium deposits have been correlated to reduced displacement levels indicative of stiffer material behavior^33^, whereas (fibro)atheromatous plaques have been associated with increasing strain levels *in-vivo*^15^. Conversely, lipid-rich lesions have been linked to decreased strain levels in previous elastography evaluations^34^, whereas in one example vulnerable lesions have been associated to levels of ‘mixed stiffness’ (defined as 26–65 kPa)^35^. The increasing group velocity standard deviation could be due to the complexity of this type class, and does accentuate the need for additional computational or experimental validation studies to causally relate specific wave propagation behavior to defined plaque composition.

Apart from the AHA type VI plaques, all other AHA types indicated fairly similar shear wave velocities. In particular, it is worth noting that the shear wave output for the non-calcified lesions (AHA type III) coincided with the output of the calcified ones (AHA type VII) – a curious finding considering that calcified deposits have been correlated to stiffer material response^33^, and how the presence of microcalcifications has been linked to local intraplaque stress/strain concentrations^36^. Instead, the results might stem from the fact that calcified regions are typically difficult to assess by means of ultrasound imaging, where pronounced calcifications induce severe shadowing artefacts and signal deterioration. The shear wave output from calcified lesions might thus be dominated by other detectable adjacent intraplaque components, maintaining the derived shear wave velocities at fairly moderate levels. Detailed computational studies may be required to elucidate the link between calcified deposits and obtained shear wave values.

### Intraplaque component correlations with shear wave velocities

A set of parameters showed correlation to intraplaque constituents. Most importantly, group velocity showed positive correlation to LRNC content, whereas both group velocity and phase velocity at the highest frequency band showed negative correlation to fibrous cap/necrotic core volume ratio –parameters associated with the identification of vulnerable TFCA. With phase velocity indicating additional unique correlations to calcification and intraplaque hemorrhage volume – parameters again tightly associated with plaque vulnerability – this exemplifies how combined SWE analysis might assist in refined plaque risk assessment. Albeit reported correlations need to be verified in larger cohorts, the results support the notion of including frequency-dependent SWE metrics when assessing spatially confined tissue. Phase velocity SWE has for cardiovascular purposes also been related to increased accuracy^18,19^, further underlining the potential of including such analysis in future refined SWE plaque characterization attempts.

Causal explanations to the observed correlations are difficult to infer, particularly due to the complex composition of atherosclerotic plaques. In principle, a large LRNC would be associated with a softer plaque, as has been indicated by microscopic evaluation^37^. However, we observe a direct relation between group velocity and LRNC content. One reason to this might be the presence of wave-guide behavior in the spatially confined plaque, or interfering confounding factors. Particularly, similar positive correlations are present for plaque cap volume, where increasing fibrous cap size and LRNC might relate to an increasing group velocity. A positive correlation is in fact apparent between LRNC content and cap volume (Pearson R = 0.80, p < 0.001), further suggesting that there might be a multifactorial explanation to the observed increase in SWE group velocity. For the phase velocity analysis, one might also question whether a thin fibrous cap could be resolved at the frequency ranges and wave speeds obtained. However, from the perspective of having a larger volume fraction of fibrous cap, it is likely that the overall stiffness of the plaque – the metric assessed by our SWE analysis – would still be affected, indirectly linking cap thickness to shear wave velocities.

For phase velocity, negative correlation was observed with the ratio between fibrous cap and necrotic core volume. With a decreasing ratio indicating a larger necrotic core at the expense of a thinner fibrous cap (indicative of TFCAs), the entity is of direct clinical importance. That increasing phase velocity is observed for such TFCA-like plaques is in-line with the increasing velocities seen in AHA type VI plaques, and would be supported by studies showing decreasing strain levels with lipid-rich core content^34^. As discussed previously however, other studies point towards the opposite with increasing strain levels having been correlated with decreasing cap thickness^14^, and where fibrous cap regions have been associated with decreasing mechanical displacement^33^. In fact, the notion that increasing intraplaque activity correlates with softer plaques is supported by the observed nonlinear negative correlation with phase velocity at 300–400 Hz and IPH. The remaining discrepancy for fibrous cap/necrotic core volume ratio might be explained by confounding factors, or from the implicit relation between the ratio and actual TFCA behavior. Similarly, it is also likely that different plaque entities influence the globally assessed plaque shear wave velocity to different extent, even though it remains to be determined whether global wave velocities are predominantly governed by stiffer or softer plaque regions. Further validation would be required to clarify the observed relationship’s causal background.

A few correlations were also observed with blood lipid levels, with HDL showing positive correlation with all evaluated wave velocities. Previous studies have reported associations between hypercholesterolemia and necrotic core volume^38^, and between non-HDL cholesterol and the prevalence of non-calcified coronary plaques^39^. For HDL previous findings indicate a negative correlation to plaque burden and LRNC volume^40^. Even though our study seem to indicate stiffer plaques with increasing HDL-levels, the specific relation between plaque stiffness and blood lipid levels remains to be evaluated in detail.

### Longitudinal and transverse plane imaging

It is worth noting that reported findings differed between longitudinal and transverse imaging plane, both with regards to plaque differentiability and observed correlations; a fact that might originate from differences in fundamental wave propagation between the two imaging planes. In longitudinal view, the shear wave propagation follows the axial vessel direction, with guided waves following the arterial and intravascular plaque structure. In comparison, in the transverse view the shear wave propagates unobstructed before traversing through the plaque as an embedded inclusion rather than as a waveguide. Thus, the two imaging planes offer different modes of wave propagation, with differences in assessed wave speed even observed in simplified *in-silico* setups^41^. Transverse plane circumferential wave guide behavior has been studied in literature^42^, however requires refined acquisition settings not used in this particular study.

### Proposed shear wave analysis vs. system-derived shear wave output

**Figure 6:**
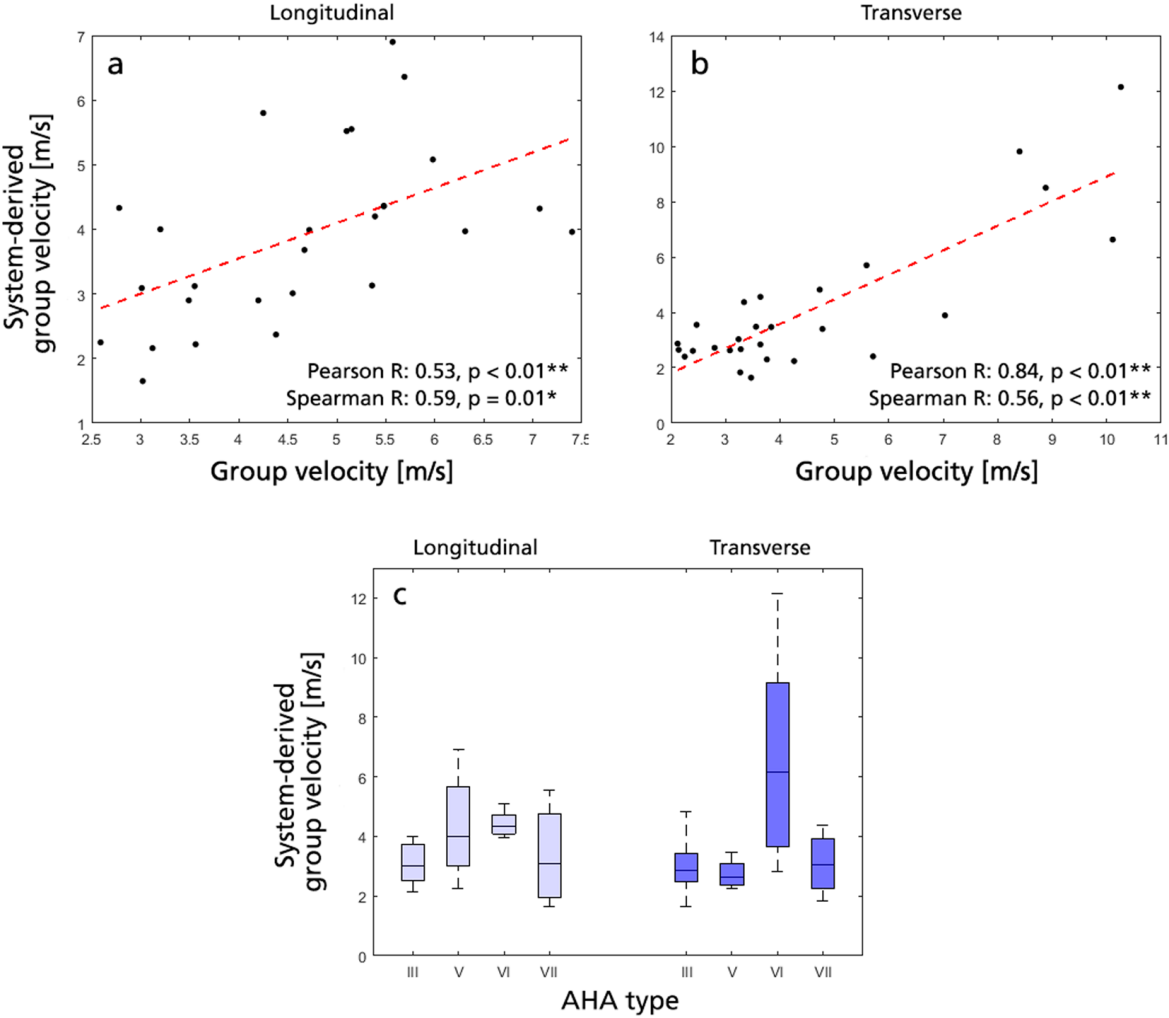
Comparison between group velocity derived using the approach described in the method section, vs. using a vendor-specific system-derived shear wave output. (**a**) Correlation plot in longitudinal view, with a linear regression slope of m = 0.55. (**b**) Correlation plot in transverse view, with a linear regression slope of m = 0.89. (**c**) Vendor-specific system-derived group velocity as a function of AHA type, where no statistical differentiation could be inferred for the AHA type VI plaques against all other evaluated plaque types, respectively.

In this study, group and phase velocity metrics were derived from acquired IQ-data using previously described processing steps, involving axial particle velocity analysis in both spatiotemporal and frequency-dependent domains^18,19^. However, to date many clinical systems – including the one utilized in this study – offer direct reconstruction of group velocity, often visualized as a superimposed color map on the clinical image display (Fig. 2, second column). As an alternative to our proposed analysis, one could thus imagine an approach where system-derived values are simply averaged within the same plaque ROI to generate group velocity outputs (as has been done in several previous studies^17,35,43^). Clinical SWE systems are however rarely calibrated for confined tissue analysis, and are instead geared towards unobstructed wave propagation through e.g. liver of breast tissue^44,45^. As such, tissue is often assumed locally homogeneous with a standardized moving window employed to reconstruct local group velocity, and in a confined plaque ROI output might be erroneously influenced by surrounding lumen or tissue signal. If comparing our derived group velocity values to averaged system-specific ones, positive correlation is observed, however with larger deviations reported in the longitudinal view (Pearson R = 0.53/0.84, p = 0.004/ < 0.001 for L/T-view, respectively, see Fig. 6(a,b)). With the longitudinal view generating a more guided wave propagation pattern (as discussed above), this might point towards the drawbacks of the vendor-specific approach, however a more systematic analysis would be required in a more controlled setup. Added to this, the separation of different AHA type plaques deteriorated when using the vendor specific direct reconstruction (see Fig. 6(c)), again underlining the benefits of our previously proposed and verified approach. Regardless, the deviation between reconstructions highlights the complexity of assessing confined atherosclerotic tissue by ultrasound elastography, and might provide a rationale to the spread in atherosclerotic shear wave velocities reported in literature^15,33–35^.

### Comparison with other imaging techniques

Compared to catheter-, MRI-, or CT-based alternatives, ultrasound SWE offers a real-time non-invasive, non-ionizing, portable and inexpensive method for plaque risk assessment. Other ultrasound-based alternatives have been proposed with compound ultrasound strain imaging^15^, non-invasive vascular elastography^15,16^, and acoustic radiation force impulse imaging^33^, all having been applied for refined plaque characterization. In comparison, SWE offers additional frequency-dependent phase velocity retrieval, with such enabling the accurate quantification of tissue shear modulus even in highly confined vascular media^19^. A quantitative comparison between ultrasound elastography methods for plaque characterization however remains to be performed.

### Limitations

This study represents a feasibility evaluation of SWE for atherosclerotic plaque characterization, and a larger cohort would be required to evaluate the method’s ability to improve clinical outcome. Increasing the number of enrolled patients would also elucidate the uncertainty and robustness of the derived metrics and correlations, especially considering the herein reported standard deviations and the previously reported technique variability^18^. Additionally, out of the 410 acquisitions, a distinct proportion had to be excluded from the phase velocity analysis due to poor dispersion quality. With frequency-domain bandwidth inversely proportional to SWE push duration^25^, the relatively long push of the utilized clinical SWE-system could explain this proportion. In future studies a decreased push duration would therefore be recommended, especially with previous studies indicating improved plaque differentiability and SWE accuracy at 1 kHz^18^. Due to technical limitations, ECG triggering was not used during acquisition, and instead multiple acquisitions were performed to average potential shear wave variations over the cardiac cycle. Since previous studies have indicated variations of up to 1.5 m/s within a single ECG cycle^46^, ECG triggering would be a recommended addition for future studies. Further potential improvements would be to evaluate intraplaque wave velocities, derive constitutive stiffness, or expand analysis to attenuating viscous behavior. However, for the former this would put increasing demand on acquisition signal quality, whereas for the latter, this would require introduced assumption on plaque acoustic and constitutive behavior, or involve modified image sequencing.

With respect to the MRI data, no spatial registration of SWE and MRI was performed, and merely global evaluation of shear wave velocity versus MRI-derived entities was performed. Similarly, 2D SWE data was compared against 3D MRI volumes, whereby a bias might be present with respect to the sonographic choice of 2D SWE image plane. Future studies using 3D SWE would be a possibility^47^, however would require using hardware and software not currently available on any clinically certified system. To this, albeit MRI has been extensively used for plaque characterization, complementary histology could have further confirmed the obtained results. However, with endarterectomy decided on a basis of patient symptoms and luminal stenosis, such data would not have been obtainable for all evaluated plaques.

## Conclusion

In this feasibility and validation study, we demonstrated for the first time that non-invasive characterization of vulnerable carotid plaque lesions can be achieved by combined group and frequency-dependent phase velocity SWE analysis. This combined SWE approach provides a set of novel imaging biomarkers correlated to vulnerable plaque features such as LRNC content, fibrous cap/necrotic core volume ratio, and IPH volume.

## Supplementary information

Supplementary material.

## Data Availability

All data generated or analysed during this study are included in this published article (and its Supplementary Information Files).
