# Author Correction: Combined spatiotemporal and frequency-dependent shear wave elastography enables detection of vulnerable carotid plaques as validated by MRI

**DOI:** 10.1038/s41598-020-69148-y

**Published:** 2020-07-17

**Authors:** David Marlevi, Sharon L. Mulvagh, Runqing Huang, J. Kevin DeMarco, Hideki Ota, John Huston, Reidar Winter, Thanila A. Macedo, Sahar S. Abdelmoneim, Matilda Larsson, Patricia A. Pellikka, Matthew W. Urban

**Affiliations:** 1 Department of Biomedical Engineering and Health Systems, KTH Royal Institute of Technology, Stockholm, Sweden; 2 Department of Clinical Sciences, Karolinska Institutet, Stockholm, Sweden; 3 Department of Cardiovascular Medicine, Mayo Clinic College of Medicine, Rochester, MN USA; 4 Division of Cardiology, Dalhousie University, Halifax, NS Canada; 5 Department of Radiology, Walter Reed National Military Medical Center, Bethesda, MD USA; 6 Department of Radiology, Uniformed Services University of Health Sciences, Bethesda, MD USA; 7 Department of Diagnostic Radiology, Tohoku University Hospital, Sendai, Japan; 8 Department of Radiology, Mayo Clinic College of Medicine, Rochester, MN USA; 9 Department of Molecular Medicine and Surgery, Karolinska Institutet, Stockholm, Sweden

Correction to: *Scientific Reports* 10.1038/s41598-019-57317-7, published online 15 January 2020

In the original version of this Article, the author J. Kevin DeMarco was incorrectly indexed. This error has now been corrected.

