# Supplementary material for: Combined spatiotemporal and frequency-dependent shear wave elastography enables detection of vulnerable carotid plaques as validated by MRI
Source: Sci Rep. 2020 Jan 15;10:403. doi: 10.1038/s41598-019-57317-7 (PMC6962347; doi:10.1038/s41598-019-57317-7)
Supplement: Supplementary file 1 — Supplementary material. [file 41598_2019_57317_MOESM1_ESM.docx]

**Combined spatiotemporal and frequency-dependent shear wave elastography enables detection of vulnerable carotid plaques as validated by MRI**

*David Marlevi^1,2,*^, Sharon L. Mulvagh^3,4^, Runqing Huang^3^, J. Kevin DeMarco^5,6^, Hideki Ota^7^,
John Huston III^8^, Reidar Winter^2^, Thanila A. Macedo^8^, Sahar S. Abdelmoneim^3^, Matilda Larsson^1,9^, Patricia A. Pellikka^3^, Matthew W. Urban^8^*

^1^Department of Biomedical Engineering and Health Systems, KTH Royal Institute of Technology, Stockholm, Sweden
^2^Department of Clinical Sciences, Karolinska Institutet, Stockholm, Sweden
^3^Department of Cardiovascular Medicine, Mayo Clinic College of Medicine, Rochester, MN, United States of America ^4^Division of Cardiology, Dalhousie University, Halifax, Nova Scotia, Canada ^5^Department of Radiology, Walter Reed National Military Medical Center, Bethesda, MD, United States of America
^6^Department of Radiology, Uniformed Services University of Health Sciences, Bethesda, MD, United States of America
^7^Department of Diagnostic Radiology, Tohoku University Hospital, Sendai, Japan
^8^Department of Radiology, Mayo Clinic College of Medicine, Rochester, MN, United States of America ^9^Department of Molecular Medicine and Surgery, Karolinska Institutet, Stockholm, Sweden

**Supplementary material**

Pearson and Spearman correlation coefficients with corresponding p-values are given in the below Supplementary tables A-D.

Table A: Pearson and Spearman correlation coefficients and p-values for the SWE group velocity analysis.

|  | Group velocity | | | | | | | |
| --- | --- | --- | --- | --- | --- | --- | --- | --- |
|  | Longitudinal | | | | Transverse | | | |
|  | Pearson | | Spearman | | Pearson | | Spearman | |
|  | R | p | R | p | R | p | R | p |
| Percentage lipid-rich necrotic core [%] | 0.57 | 0.02 | 0.63 | 0.01 | 0.58 | 0.02 | 0.63 | 0.01 |
| Percentage loose  matrix [%] | -0.29 | 0.36 | -0.29 | 0.37 | -0.55 | 0.07 | -0.48 | 0.12 |
| Plaque length | 0.14 | 0.51 | 0.20 | 0.34 | 0.39 | 0.05 | 0.17 | 0.42 |
| Plaque thickness | 0.14 | 0.51 | 0.11 | 0.59 | 0.16 | 0.44 | 0.08 | 0.71 |
| Plaque area | 0.03 | 0.90 | 0.03 | 0.88 | 0.32 | 0.12 | 0.12 | 0.57 |
| Max. necrotic/lipid core area | 0.39 | 0.13 | 0.44 | 0.09 | 0.52 | 0.04 | 0.61 | 0.01 |
| Percentage calcification [%] | -0.50 | 0.10 | -0.46 | 0.13 | -0.33 | 0.30 | -0.20 | 0.54 |
| Percentage IPH [%] | -0.44 | 0.39 | -0.49 | 0.36 | -0.25 | 0.63 | -0.71 | 0.14 |
| Max. loose matrix area | -0.12 | 0.72 | -0.20 | 0.54 | -0.25 | 0.43 | 0.03 | 0.93 |
| Necrotic core volume | 0.60 | 0.01 | 0.67 | 0.01 | 0.62 | 0.01 | 0.66 | 0.01 |
| Calcification volume | -0.22 | 0.49 | -0.28 | 0.38 | 0.03 | 0.92 | -0.06 | 0.87 |
| Hemorrhage volume | -0.46 | 0.36 | -0.60 | 0.24 | -0.23 | 0.66 | -0.60 | 0.24 |
| Loose matrix volume | -0.20 | 0.54 | -0.22 | 0.49 | -0.44 | 0.15 | -0.41 | 0.19 |
| Fibrous cap volume | 0.60 | 0.02 | 0.67 | 0.01 | 0.46 | 0.07 | 0.55 | 0.03 |
| Plaque wall volume | 0.38 | 0.05 | 0.41 | 0.04 | 0.41 | 0.04 | 0.48 | 0.01 |
| Max normalized wall index (NWI) | -0.01 | 0.98 | 0.01 | 0.97 | 0.29 | 0.16 | 0.11 | 0.60 |
| Ratio: loose matrix area / Max. NWI | -0.06 | 0.85 | -0.20 | 0.54 | -0.30 | 0.34 | -0.07 | 0.82 |
| Ratio: loose matrix / Max. NWI | -0.06 | 0.85 | -0.20 | 0.54 | -0.30 | 0.34 | -0.07 | 0.82 |
| Percentage fibrous cap volume [%] | 0.58 | 0.02 | 0.63 | 0.01 | 0.41 | 0.11 | 0.50 | 0.05 |
| Ratio: Fibrous cap volume / Necrotic core volume | -0.21 | 0.43 | -0.43 | 0.10 | -0.46 | 0.07 | -0.61 | 0.01 |
| Fibrous cap area | 0.37 | 0.15 | 0.47 | 0.06 | 0.12 | 0.66 | 0.37 | 0.15 |
| Max. fibrous cap thickness | 0.48 | 0.05 | 0.45 | 0.07 | 0.20 | 0.45 | 0.33 | 0.20 |
| Min. fibrous cap thickness | 0.16 | 0.53 | -0.25 | 0.34 | 0.26 | 0.32 | -0.16 | 0.55 |
| Mean fibrous cap thickness | 0.25 | 0.33 | 0.06 | 0.83 | 0.31 | 0.23 | 0.00 | 1.00 |
| Fibrous cap length | 0.23 | 0.38 | 0.28 | 0.28 | 0.32 | 0.21 | 0.51 | 0.04 |
| Age | 0.01 | 0.96 | -0.02 | 0.91 | -0.18 | 0.38 | -0.26 | 0.20 |
| BMI | -0.22 | 0.28 | -0.20 | 0.32 | -0.39 | 0.05 | -0.19 | 0.35 |
| Total cholesterol | 0.04 | 0.86 | 0.14 | 0.48 | -0.09 | 0.65 | -0.06 | 0.78 |
| Triglycerides (TG) | 0.02 | 0.94 | -0.06 | 0.79 | -0.27 | 0.18 | -0.20 | 0.32 |
| High-density lipoprotein (HDL) | 0.42 | 0.03 | 0.36 | 0.08 | 0.56 | 0.00 | 0.43 | 0.03 |
| Low-denisty lipoprotein (LDL) | -0.14 | 0.49 | -0.09 | 0.65 | -0.18 | 0.37 | -0.21 | 0.30 |
| Total non-HDL cholesterol | -0.12 | 0.59 | -0.02 | 0.92 | -0.31 | 0.18 | -0.33 | 0.15 |
| Ratio: LDL/HDL | -0.34 | 0.09 | -0.30 | 0.14 | -0.38 | 0.06 | -0.45 | 0.02 |
| Ratio: HDL/Total cholesterol | 0.24 | 0.25 | 0.26 | 0.19 | 0.50 | 0.01 | 0.43 | 0.03 |
| Ratio: LDL/Total cholesterol | -0.26 | 0.21 | -0.24 | 0.24 | -0.22 | 0.27 | -0.27 | 0.18 |
| Ratio: TG/HDL | -0.05 | 0.81 | -0.19 | 0.35 | -0.31 | 0.12 | -0.34 | 0.09 |
| Ratio: TG/LDL | 0.13 | 0.54 | 0.02 | 0.93 | -0.14 | 0.49 | -0.10 | 0.62 |
| Blood glucose | 0.07 | 0.74 | -0.31 | 0.13 | -0.19 | 0.35 | -0.35 | 0.08 |
| Systolic blood pressure (SBP) | -0.09 | 0.67 | 0.05 | 0.79 | 0.00 | 0.98 | -0.10 | 0.63 |
| Diastolic blood pressure (DBP) | 0.00 | 0.99 | 0.12 | 0.56 | 0.07 | 0.73 | 0.08 | 0.69 |

Table B: Pearson and Spearman correlation coefficients and p-values for the SWE phase velocity analysis at 200-300 Hz.

|  | Phase velocity, 200-300 Hz | | | | | | | |
| --- | --- | --- | --- | --- | --- | --- | --- | --- |
|  | Longitudinal | | | | Transverse | | | |
|  | Pearson | | Spearman | | Pearson | | Spearman | |
|  | R | p | R | p | R | p | R | p |
| Percentage lipid-rich necrotic core [%] | 0.21 | 0.43 | 0.12 | 0.66 | 0.26 | 0.36 | 0.24 | 0.38 |
| Percentage loose  matrix [%] | 0.16 | 0.61 | 0.18 | 0.57 | -0.54 | 0.07 | -0.50 | 0.10 |
| Plaque length | 0.04 | 0.86 | 0.12 | 0.56 | 0.10 | 0.64 | 0.10 | 0.65 |
| Plaque thickness | -0.20 | 0.32 | -0.12 | 0.57 | 0.28 | 0.18 | 0.18 | 0.38 |
| Plaque area | -0.05 | 0.82 | 0.01 | 0.98 | 0.10 | 0.64 | 0.24 | 0.27 |
| Max. necrotic/lipid core area | -0.05 | 0.87 | -0.13 | 0.63 | 0.36 | 0.18 | 0.33 | 0.23 |
| Percentage calcification [%] | -0.23 | 0.48 | -0.22 | 0.50 | -0.10 | 0.78 | -0.24 | 0.49 |
| Percentage IPH [%] | 0.20 | 0.70 | -0.09 | 0.92 | -0.26 | 0.68 | -0.10 | 0.95 |
| Max. loose matrix area | -0.19 | 0.56 | -0.21 | 0.51 | -0.03 | 0.93 | 0.15 | 0.65 |
| Necrotic core volume | 0.25 | 0.35 | 0.12 | 0.66 | 0.24 | 0.38 | 0.25 | 0.38 |
| Calcification volume | -0.16 | 0.61 | -0.12 | 0.72 | 0.02 | 0.97 | -0.07 | 0.84 |
| Hemorrhage volume | 0.09 | 0.87 | -0.31 | 0.56 | -0.33 | 0.59 | -0.20 | 0.78 |
| Loose matrix volume | 0.08 | 0.80 | 0.07 | 0.83 | -0.44 | 0.15 | -0.36 | 0.26 |
| Fibrous cap volume | 0.09 | 0.74 | 0.06 | 0.82 | 0.01 | 0.98 | 0.03 | 0.93 |
| Plaque wall volume | 0.03 | 0.87 | -0.10 | 0.63 | 0.27 | 0.19 | 0.35 | 0.08 |
| Max normalized wall index (NWI) | 0.16 | 0.45 | 0.31 | 0.15 | 0.12 | 0.59 | -0.03 | 0.90 |
| Ratio: loose matrix area / Max. NWI | -0.05 | 0.87 | -0.31 | 0.33 | -0.08 | 0.82 | -0.18 | 0.59 |
| Ratio: loose matrix / Max. NWI | -0.05 | 0.87 | -0.31 | 0.33 | -0.08 | 0.82 | -0.18 | 0.59 |
| Percentage fibrous cap volume [%] | 0.07 | 0.80 | 0.11 | 0.69 | -0.03 | 0.92 | 0.01 | 0.97 |
| Ratio: Fibrous cap volume / Necrotic core volume | -0.15 | 0.58 | -0.22 | 0.40 | -0.50 | 0.06 | -0.61 | 0.02 |
| Fibrous cap area | -0.27 | 0.30 | -0.13 | 0.61 | -0.15 | 0.59 | -0.12 | 0.67 |
| Max. fibrous cap thickness | 0.11 | 0.68 | 0.02 | 0.95 | -0.02 | 0.94 | 0.02 | 0.93 |
| Min. fibrous cap thickness | 0.22 | 0.41 | 0.21 | 0.41 | -0.18 | 0.51 | -0.10 | 0.72 |
| Mean fibrous cap thickness | 0.13 | 0.61 | 0.16 | 0.55 | -0.14 | 0.60 | -0.10 | 0.70 |
| Fibrous cap length | -0.33 | 0.20 | -0.24 | 0.35 | 0.20 | 0.47 | 0.30 | 0.25 |
| Age | 0.06 | 0.76 | 0.00 | 0.98 | -0.15 | 0.46 | -0.26 | 0.20 |
| BMI | -0.01 | 0.95 | -0.05 | 0.79 | 0.06 | 0.78 | 0.03 | 0.89 |
| Total cholesterol | -0.10 | 0.63 | 0.04 | 0.87 | -0.38 | 0.06 | -0.40 | 0.04 |
| Triglycerides (TG) | -0.01 | 0.96 | -0.12 | 0.57 | -0.43 | 0.03 | -0.40 | 0.03 |
| High-density lipoprotein (HDL) | 0.15 | 0.46 | 0.28 | 0.16 | 0.59 | 0.002 | 0.54 | 0.004 |
| Low-denisty lipoprotein (LDL) | -0.17 | 0.40 | -0.08 | 0.71 | -0.40 | 0.04 | -0.35 | 0.08 |
| Total non-HDL cholesterol | -0.15 | 0.51 | -0.10 | 0.67 | -0.64 | 0.002 | -0.69 | 0.001 |
| Ratio: LDL/HDL | -0.23 | 0.27 | -0.23 | 0.27 | -0.59 | 0.002 | -0.66 | 0.001 |
| Ratio: HDL/Total cholesterol | 0.18 | 0.39 | 0.25 | 0.23 | 0.71 | 0.001 | 0.69 | 0.001 |
| Ratio: LDL/Total cholesterol | -0.21 | 0.30 | -0.13 | 0.54 | -0.32 | 0.10 | -0.33 | 0.10 |
| Ratio: TG/HDL | -0.06 | 0.77 | -0.19 | 0.36 | -0.43 | 0.03 | -0.60 | 0.001 |
| Ratio: TG/LDL | 0.08 | 0.70 | -0.11 | 0.60 | -0.21 | 0.30 | -0.21 | 0.31 |
| Blood glucose | -0.05 | 0.83 | -0.34 | 0.09 | -0.36 | 0.08 | -0.50 | 0.01 |
| Systolic blood pressure (SBP) | 0.02 | 0.93 | 0.10 | 0.64 | 0.06 | 0.78 | 0.08 | 0.70 |
| Diastolic blood pressure (DBP) | -0.19 | 0.34 | -0.16 | 0.43 | 0.29 | 0.16 | 0.32 | 0.11 |

Table C: Pearson and Spearman correlation coefficients and p-values for the SWE phase velocity analysis at 300-400 Hz.

|  | Phase velocity, 300-400 Hz | | | | | | | |
| --- | --- | --- | --- | --- | --- | --- | --- | --- |
|  | Longitudinal | | | | Transverse | | | |
|  | Pearson | | Spearman | | Pearson | | Spearman | |
|  | R | p | R | p | R | p | R | p |
| Percentage lipid-rich necrotic core [%] | 0.33 | 0.21 | 0.14 | 0.61 | 0.39 | 0.16 | 0.26 | 0.35 |
| Percentage loose  matrix [%] | -0.25 | 0.44 | -0.18 | 0.57 | -0.65 | 0.02 | -0.63 | 0.03 |
| Plaque length | 0.13 | 0.53 | 0.26 | 0.19 | 0.39 | 0.05 | 0.20 | 0.34 |
| Plaque thickness | -0.06 | 0.79 | 0.01 | 0.97 | 0.31 | 0.13 | 0.27 | 0.20 |
| Plaque area | 0.03 | 0.89 | 0.05 | 0.82 | 0.37 | 0.08 | 0.22 | 0.30 |
| Max. necrotic/lipid core area | -0.07 | 0.80 | -0.07 | 0.81 | 0.36 | 0.19 | 0.30 | 0.28 |
| Percentage calcification [%] | -0.24 | 0.45 | -0.17 | 0.60 | 0.31 | 0.35 | 0.44 | 0.18 |
| Percentage IPH [%] | -0.74 | 0.10 | -1.00 | 0.00 | -0.03 | 0.96 | -0.70 | 0.23 |
| Max. loose matrix area | -0.26 | 0.41 | -0.28 | 0.38 | -0.18 | 0.58 | 0.01 | 0.99 |
| Necrotic core volume | 0.37 | 0.15 | 0.19 | 0.47 | 0.45 | 0.10 | 0.29 | 0.30 |
| Calcification volume | -0.16 | 0.63 | -0.02 | 0.96 | 0.70 | 0.02 | 0.59 | 0.06 |
| Hemorrhage volume | -0.72 | 0.11 | -0.94 | 0.02 | 0.02 | 0.98 | -0.40 | 0.52 |
| Loose matrix volume | -0.12 | 0.71 | -0.03 | 0.94 | -0.56 | 0.06 | -0.50 | 0.10 |
| Fibrous cap volume | 0.36 | 0.18 | 0.16 | 0.56 | 0.25 | 0.37 | 0.17 | 0.54 |
| Plaque wall volume | 0.20 | 0.32 | 0.13 | 0.53 | 0.32 | 0.12 | 0.28 | 0.18 |
| Max normalized wall index (NWI) | 0.11 | 0.60 | -0.01 | 0.98 | 0.33 | 0.13 | 0.18 | 0.40 |
| Ratio: loose matrix area / Max. NWI | 0.03 | 0.94 | -0.17 | 0.60 | -0.23 | 0.47 | -0.28 | 0.38 |
| Ratio: loose matrix / Max. NWI | 0.03 | 0.94 | -0.17 | 0.60 | -0.23 | 0.47 | -0.28 | 0.38 |
| Percentage fibrous cap volume [%] | 0.34 | 0.20 | 0.15 | 0.59 | 0.16 | 0.58 | 0.06 | 0.84 |
| Ratio: Fibrous cap volume / Necrotic core volume | -0.11 | 0.70 | -0.16 | 0.56 | -0.40 | 0.14 | -0.41 | 0.13 |
| Fibrous cap area | 0.04 | 0.87 | 0.04 | 0.87 | -0.03 | 0.91 | -0.06 | 0.82 |
| Max. fibrous cap thickness | 0.15 | 0.56 | -0.02 | 0.95 | -0.03 | 0.92 | 0.01 | 0.98 |
| Min. fibrous cap thickness | 0.34 | 0.18 | 0.10 | 0.71 | -0.22 | 0.42 | -0.06 | 0.83 |
| Mean fibrous cap thickness | 0.42 | 0.10 | 0.03 | 0.91 | 0.09 | 0.73 | 0.02 | 0.94 |
| Fibrous cap length | -0.15 | 0.57 | -0.20 | 0.43 | 0.26 | 0.33 | 0.27 | 0.32 |
| Age | -0.09 | 0.66 | -0.10 | 0.60 | -0.36 | 0.07 | -0.50 | 0.01 |
| BMI | -0.26 | 0.20 | -0.06 | 0.76 | -0.31 | 0.12 | -0.28 | 0.16 |
| Total cholesterol | -0.16 | 0.45 | -0.05 | 0.83 | -0.22 | 0.28 | -0.25 | 0.22 |
| Triglycerides (TG) | -0.05 | 0.81 | -0.19 | 0.36 | -0.39 | 0.05 | -0.39 | 0.05 |
| High-density lipoprotein (HDL) | 0.33 | 0.10 | 0.39 | 0.05 | 0.63 | 0.001 | 0.48 | 0.01 |
| Low-denisty lipoprotein (LDL) | -0.27 | 0.18 | -0.12 | 0.55 | -0.26 | 0.20 | -0.20 | 0.32 |
| Total non-HDL cholesterol | -0.26 | 0.25 | -0.20 | 0.38 | -0.45 | 0.04 | -0.46 | 0.04 |
| Ratio: LDL/HDL | -0.37 | 0.06 | -0.37 | 0.06 | -0.47 | 0.02 | -0.46 | 0.02 |
| Ratio: HDL/Total cholesterol | 0.32 | 0.12 | 0.40 | 0.04 | 0.62 | 0.001 | 0.53 | 0.01 |
| Ratio: LDL/Total cholesterol | -0.30 | 0.13 | -0.21 | 0.32 | -0.23 | 0.26 | -0.25 | 0.23 |
| Ratio: TG/HDL | -0.12 | 0.56 | -0.32 | 0.12 | -0.39 | 0.05 | -0.53 | 0.01 |
| Ratio: TG/LDL | 0.05 | 0.80 | -0.08 | 0.70 | -0.24 | 0.25 | -0.24 | 0.24 |
| Blood glucose | -0.15 | 0.48 | -0.54 | 0.01 | -0.34 | 0.10 | -0.37 | 0.07 |
| Systolic blood pressure (SBP) | 0.04 | 0.84 | 0.21 | 0.30 | 0.20 | 0.33 | 0.13 | 0.54 |
| Diastolic blood pressure (DBP) | -0.08 | 0.70 | 0.06 | 0.78 | 0.41 | 0.04 | 0.41 | 0.04 |

Table D: Pearson and Spearman correlation coefficients and p-values for the SWE phase velocity analysis at 400-500 Hz.

|  | Phase velocity, 400-500 Hz | | | | | | | |
| --- | --- | --- | --- | --- | --- | --- | --- | --- |
|  | Longitudinal | | | | Transverse | | | |
|  | Pearson | | Spearman | | Pearson | | Spearman | |
|  | R | p | R | p | R | p | R | p |
| Percentage lipid-rich necrotic core [%] | 0.27 | 0.34 | 0.367 | 0.274 | 0.28 | 0.31 | 0.28 | 0.31 |
| Percentage loose  matrix [%] | -0.37 | 0.24 | -0.47 | -0.37 | -0.64 | 0.03 | -0.57 | 0.06 |
| Plaque length | 0.45 | 0.03 | 0.50 | 0.45 | 0.18 | 0.38 | -0.02 | 0.94 |
| Plaque thickness | 0.08 | 0.70 | 0.07 | 0.083 | 0.24 | 0.24 | 0.20 | 0.33 |
| Plaque area | 0.12 | 0.59 | 0.09 | 0.12 | 0.18 | 0.40 | 0.03 | 0.88 |
| Max. necrotic/lipid core area | 0.34 | 0.2 | 0.32 | 0.34 | 0.65 | 0.01 | 0.60 | 0.02 |
| Percentage calcification [%] | -0.44 | 0.18 | -0.29 | -0.44 | 0.34 | 0.31 | 0.42 | 0.20 |
| Percentage IPH [%] | -0.51 | 0.38 | -0.60 | -0.51 | -0.37 | 0.54 | -0.70 | 0.23 |
| Max. loose matrix area | -0.21 | 0.51 | -0.11 | -0.21 | -0.10 | 0.76 | 0.11 | 0.73 |
| Necrotic core volume | 0.36 | 0.21 | 0.42 | 0.36 | 0.25 | 0.37 | 0.26 | 0.35 |
| Calcification volume | 0.08 | 0.82 | 0.04 | 0.08 | 0.64 | 0.04 | 0.51 | 0.11 |
| Hemorrhage volume | -0.50 | 0.39 | -0.30 | -0.50 | -0.34 | 0.58 | -0.40 | 0.52 |
| Loose matrix volume | -0.18 | 0.57 | -0.24 | -0.18 | -0.46 | 0.13 | -0.41 | 0.19 |
| Fibrous cap volume | 0.19 | 0.53 | 0.35 | 0.19 | -0.03 | 0.91 | 0.06 | 0.83 |
| Plaque wall volume | 0.36 | 0.09 | 0.35 | 0.36 | 0.25 | 0.23 | 0.20 | 0.35 |
| Max normalized wall index (NWI) | -0.17 | 0.46 | -0.18 | -0.17 | 0.40 | 0.06 | 0.18 | 0.41 |
| Ratio: loose matrix area / Max. NWI | 0.18 | 0.58 | -0.06 | 0.18 | -0.16 | 0.63 | -0.17 | 0.60 |
| Ratio: loose matrix / Max. NWI | 0.18 | 0.58 | -0.06 | 0.18 | -0.16 | 0.63 | -0.17 | 0.60 |
| Percentage fibrous cap volume [%] | 0.03 | 0.91 | 0.14 | 0.03 | -0.05 | 0.87 | -0.05 | 0.87 |
| Ratio: Fibrous cap volume / Necrotic core volume | -0.28 | 0.33 | -0.46 | -0.28 | -0.49 | 0.06 | -0.61 | 0.02 |
| Fibrous cap area | -0.05 | 0.85 | 0.05 | -0.05 | -0.11 | 0.69 | -0.13 | 0.64 |
| Max. fibrous cap thickness | -0.13 | 0.65 | -0.02 | -0.13 | -0.29 | 0.28 | -0.13 | 0.63 |
| Min. fibrous cap thickness | -0.24 | 0.39 | -0.22 | -0.24 | -0.26 | 0.33 | -0.10 | 0.70 |
| Mean fibrous cap thickness | -0.40 | 0.15 | -0.34 | -0.40 | -0.31 | 0.25 | -0.32 | 0.22 |
| Fibrous cap length | 0.04 | 0.89 | 0.13 | 0.04 | 0.46 | 0.07 | 0.50 | 0.05 |
| Age | -0.07 | 0.76 | -0.16 | 0.43 | -0.44 | 0.02 | -0.55 | 0.004 |
| BMI | -0.14 | 0.50 | -0.18 | 0.38 | -0.20 | 0.33 | -0.21 | 0.31 |
| Total cholesterol | -0.06 | 0.78 | 0.06 | 0.78 | -0.25 | 0.21 | -0.35 | 0.08 |
| Triglycerides (TG) | -0.20 | 0.34 | -0.27 | 0.20 | -0.52 | 0.01 | -0.52 | 0.01 |
| High-density lipoprotein (HDL) | 0.57 | 0.003 | 0.54 | 0.01 | 0.52 | 0.01 | 0.39 | 0.05 |
| Low-denisty lipoprotein (LDL) | -0.17 | 0.43 | -0.07 | 0.73 | -0.18 | 0.39 | -0.17 | 0.41 |
| Total non-HDL cholesterol | -0.29 | 0.22 | -0.24 | 0.31 | -0.43 | 0.05 | -0.50 | 0.02 |
| Ratio: LDL/HDL | -0.39 | 0.06 | -0.46 | 0.02 | -0.35 | 0.08 | -0.38 | 0.06 |
| Ratio: HDL/Total cholesterol | 0.51 | 0.01 | 0.49 | 0.01 | 0.60 | 0.001 | 0.52 | 0.01 |
| Ratio: LDL/Total cholesterol | -0.20 | 0.35 | -0.20 | 0.34 | -0.06 | 0.76 | -0.10 | 0.65 |
| Ratio: TG/HDL | -0.25 | 0.22 | -0.43 | 0.03 | -0.45 | 0.02 | -0.60 | 0.001 |
| Ratio: TG/LDL | -0.11 | 0.60 | -0.19 | 0.37 | -0.38 | 0.05 | -0.34 | 0.09 |
| Blood glucose | -0.13 | 0.55 | -0.49 | 0.02 | -0.43 | 0.03 | -0.38 | 0.06 |
| Systolic blood pressure (SBP) | 0.36 | 0.07 | 0.49 | 0.01 | -0.04 | 0.85 | -0.09 | 0.65 |
| Diastolic blood pressure (DBP) | 0.37 | 0.07 | 0.47 | 0.02 | 0.21 | 0.32 | 0.20 | 0.33 |
